# Commentary: Patient Cost Sharing and Medical Expenditures for the Elderly

**DOI:** 10.3389/fphar.2016.00073

**Published:** 2016-03-30

**Authors:** Mihajlo Jakovljevic

**Affiliations:** Graduate Health Economics and Pharmacoeconomics Curricula, Faculty of Medical Sciences, University of KragujevacKragujevac, Serbia

**Keywords:** cost sharing, medical expenditure, elderly, Japan, population aging

Fukushima et al. recently published research on the relationship between cost sharing policies and medical spending by the elderly (Fukushima et al., 2016). Japan, as the nation in the most advanced stage of population aging, is indeed the best place to search for answers (Ogura et al., 2007). The authors provided us with a valuable contribution on the effects of such policies on the demand for medical services and costs within the subpopulation of senior citizens. The study has been conducted within a sound methodological framework and significantly expands our knowledge on the oldest among the world's large nations. I would like to complement their revealing findings with few additional facts crucial for understanding these issues.

One of the baseline results of the study claims: “lower cost sharing significantly increases medical spending.” This actually follows the natural logic that a patient is likely to consume more care if it is effectively free at the point of usage. Three decades ago policy makers believed it should be possible for a cost sharing mechanism to contain costs and induce net savings (Keeler and Rolph, 1983). It appears that the final balance is highly dependent on prevailing governing practices and legislative framework within the observed market (Remler and Glied, 2006). Besides there are studies showing that increased co-payments—especially for pharmaceuticals—have a detrimental effect on access and compliance—potentially leading to worse health outcomes (Shrank et al., 2006; Roberts et al., 2012a,b; Maimaris et al., 2013; Barnieh et al., 2014; Putrik et al., 2014; Simoens and Sinnaeve, 2014; Barbui and Conti, 2015; Tefferi et al., 2015). There are findings from Greece where major increases in patient co-payments were coupled with a reduction in public services in areas such as infection and mental health. This national case also adds to the literature (Ayuso-Mateos et al., 2013; Siskou et al., 2013; Kentikelenis et al., 2014). However, the impact of increased co-payments on health is still subject to ongoing debate (Mann et al., 2014). WHO had serious concerns on accessibility and co-payment. Therefore, it settled on a target of 80% availability for affordable essential medicines, including generics. Targeted therapeutic areas were major Non-Communicable Diseases (NCDs), such as diabetes and hypertension. This policy aimed to address global concerns with the impact of NCDs on morbidity and mortality^1^.

The article conveys an important message with regards to the role of cost sharing in prescribing and dispensing medicines (Johnson et al., 1997). The authors noticed the concerning fact that reduced cost sharing after the age of 70 increases demand for brand-name medicines, unlike generics. This change ultimately leads to an expanding market share of original drug dispensing and sales. The roots of this change could be found in consumer behavior. There is traditionally a strong lack of confidence in the quality of copycat medicines attributed to the Japanese patient (Kobayashi et al., 2011). This explains the willingness to pay slightly more for an original drug compared to generic alternative in a reduced cost-sharing setting. The uniqueness of the Japanese pharmaceutical market and its global impact refers to its mammoth size (World's second largest) and the smallest share of generics compared to other major high-income OECD economies (Penner-Hahn and Shaver, 2005). National efforts to increase generic replacement of brand name drugs have long been a source of public debate among Japanese authorities (Jakovljevic et al., 2014). The surprising findings of Fukushima et al. appropriately indicate the need for differential cost sharing rates as a strategy to contain drug acquisition costs in future.

There is a variety of measures used to increase the prescribing of generics vs. originators in Europe. These include compulsory substitution, e.g., in Sweden (Andersson et al., 2005), compulsory INN prescribing, e.g., in Lithuania (Garuoliene et al., 2011), or high voluntary INN prescribing as in the UK (Godman et al., 2013). The high use of generics should not be troubled conditional to guaranteed quality. Some of these historical experiences might be applicable to Japanese policy challenges as well. We should be aware that a general provision of guidelines has limited impact visible in the demise of the RMO guidelines in France for GPs (Sermet et al., 2010). The comprehensive approach to instigating a limited list of well-proven medicines, coupled with simple advice, has worked well in Stockholm, Sweden. It leads to improved care through consistency in use of well-proven medicines as well as reduced pharmaceutical expenditure (Gustafsson et al., 2011).

The phenomenon of a shrinking labor force and threatened financial sustainability of health care provision was recognized in the Japanese market a few decades ago (Ogura, 1994). The development of policies aimed at meeting this challenge followed, with diverse success rates (Tsutsui and Muramatsu, 2007; Campbell, 2014). It has clearly been proven that the long term health expenditure pattern follows the pace of population aging (Stojkovic and Milovanovic, 2015). Outsourcing from Japanese regression discontinuity study there are valuable lessons with implications for other world regions. Nations of the European region age rapidly while key consequences for the national health sectors and economies are yet to be seen (Jakovljevic M. 2015). The pace of the process remains uneven but surprisingly, involves some of the traditional young nations historically famous for their high fertility rates (Jakovljevic and Laaser, 2015). Appliance of cost sharing approaches as a strategy to contain medical costs has become prominently popular even among Eastern European countries (Tambor et al., 2015). Administration of cost sharing as demand side intervention to limit medical consumption among the elderly is clearly beneficial for the society. There remain two essential issues likely to spark further professional debate coming from opposite angles of different stakeholders. Patient perspective focuses on how to protect senior citizens from vulnerabilities outsourcing from such policies. These citizens face falling household income after retirement and lower affordability of medicines and medical services (Miralles and Kimberlin, 1998). Such financial burden is lessened by generous provision of cost sharing after the age of 70 provided in Japanese health system. Another issue comes from the perspective of the society. How to limit the heavy burden of medical expenditure attributable to the elderly within the existing financial constraints of a large aging nation? (Ogura and Jakovljevic, 2014) Strategies complementing the evident success of cost sharing will be sought in the upcoming decades as expenditure continues to grow further (Jakovljevic M. M. 2016). The advanced stage of Japanese social care for the elderly shall probably remain a prime example of the rapidly evolving health systems of the Asia-Pacific region.

## Author contributions

MJ has solely designed and authored this Commentary article without any external engagements by other contributor or professional services.

### Conflict of interest statement

The author declares that the research was conducted in the absence of any commercial or financial relationships that could be construed as a potential conflict of interest.

## Footnotes

^1^Global Action Plan for the Prevention and Control of Noncommunicable Diseases 2013–2020. http://www.who.int/nmh/events/2013/revised_draft_ncd_action_plan.pdf?ua=1 (updated revised draft).
